# Associated Factor and Long-Term Clinical Outcomes for Patients with Postoperative Rotational Malreduction in Pediatric Supracondylar Humeral Fractures

**DOI:** 10.3390/medicina60050791

**Published:** 2024-05-09

**Authors:** Kyu Bum Seo, Byung Suk Kim, Yong-Geun Park, Chaemoon Lim

**Affiliations:** Department of Orthopedic Surgery, Jeju National University Hospital, Jeju 63241, Republic of Korea; cbnuoskbs@naver.com (K.B.S.); illseat88@gmail.com (B.S.K.); pyk184@hanmail.net (Y.-G.P.)

**Keywords:** supracondylar humeral fractures, rotational malreduction, axial plane

## Abstract

*Background and Objectives*: Long-term outcomes of immediately postoperative rotational malreduction in the axial plane after operative treatment of supracondylar humeral fractures (SCHF) are unknown. This study aimed to investigate the long-term clinical outcomes and associated factors for immediately postoperative rotational malreduction of SCHF. *Materials and methods*: In this retrospective case–control study, 88 patients who underwent surgery for Gratland type III SCHF were enrolled between January 2012 and January 2020. Among them, 49 patients had immediately postoperative malrotational reduction (rotational malreduction group) and 39 patients had no rotational deformity (control group). To evaluate the associated factors for immediately postoperative rotational malreduction, demographic data, fracture patterns, physical examination signs, and preoperative radiological parameters were analyzed. To compare the clinical outcomes, operation time, range of motion of the elbow, time from operation to full range of motion, and Flynn criteria were evaluated. The Oxford elbow score was used to investigate long-term clinical outcomes for patients five years after operation. *Results*: The mean age was 5.7 ± 2.3 years and mean follow-up period was 15.7 ± 4.0 months. The rotational malreduction group had significantly more patients with oblique fracture pattern (*p* = 0.031) and Pucker sign (*p* = 0.016) and showed a significantly longer operative time (*p* = 0.029) than the control group. Although there was no significant difference in the range of elbow motion and the Flynn criteria, the Kaplan–Meier survival curve showed a longer time to recover the full range of elbow motion in the rotational malreduction group (*p* = 0.040). There were no significant differences in the long-term clinical outcomes assessed using the Oxford elbow score (*p* = 0.684). *Conclusions*: Oblique fracture pattern and Pucker sign may be associated with immediately postoperative rotational malreduction in the axial plane. Although patients with immediately postoperative rotational malreduction showed favorable results of long-term clinical outcomes, they required more weeks to recover the full range of elbow motion.

## 1. Introduction

Supracondylar humeral fractures (SCHF) are the most common elbow fractures in children with a reported prevalence of 12–17% among all pediatric fractures [1,2]. The treatment of SCHF is decided based on the Gartland classification, angulation, and associated injury [3]. Non-displaced Gartland type I SCHF can be treated using nonoperative methods [4]. Although the optimal treatment for Gartland type II SCHF remains controversial, most Gartland type III SCHF are treated surgically [4,5,6]. The displaced SCHF is treated via closed or open reduction with percutaneous pinning [7].

After the surgery, the reduction status of SCHF can be assessed using radiologic imaging. The Baumann’s angle assesses the reduction in the coronal plane, and the humerocapitellar angle helps determine the reduction in the sagittal plane [8,9]. The rotational angle assesses the correct reduction in the axial plane. Although remodeling of the coronal plane malreduction is less reliable, remodeling of the sagittal plane malreduction is believed to be possible [10,11]. The spontaneous correction of rotational malreduction in the axial plane is also believed to be possible [11]. However, persistent rotational malreduction might lead to tilting of the distal fragment into a cubitus varus deformity, which is the most common complication [12]. However, another study reported that rotational malreduction in the axial plane was not associated with cubitus varus deformities [13].

Although there are many studies on SCHF, information on the clinical and radiologic outcomes of rotational malreduction in the axial plane is limited. Several studies reported no relationship between rotational malreduction and clinical outcomes [10]. Moreover, long-term outcomes of rotational malreduction in the axial plane remain unknown. Therefore, this study aimed to investigate the long-term clinical outcomes and associated factors for immediately postoperative rotational malreduction in the axial plane after operative treatment of SCHF.

## 2. Methods

This retrospective case–control study was approved by our Institutional Review Board and all methods were performed in accordance with the guidelines and regulations of the Declaration of Helsinki. The requirement for informed consent was waived by the Institutional Review Board because of the retrospective nature of the study.

A total of 295 patients who were diagnosed with supracondylar humeral fractures between January 2012 and January 2020 were assessed. The inclusion criteria were as follows: (i) modified Gartland type III or IV SCHF requiring surgery, (ii) age < 16 years at the time of surgery, and (iii) available medical and radiological records. The exclusion criteria were as follows: (i) combined fracture of the forearm and (ii) follow-up period of <1 year. Thus, 88 patients were finally included in this study.

Two main surgeons (KBS and CL) performed the surgery. The surgical treatment of the supracondylar humeral fractures was performed under general anesthesia. All the fractures were reduced using the open or closed method. The fractures were percutaneously fixed using two or three 0.062-inch K-wires. Generally, two K-wires are inserted laterally, and a third K-wire is inserted medially or laterally, depending on stability. After surgery, the elbow was immobilized under neutral rotation and at 45–60° flexion with a splint. All patients visited the outpatient clinic at postoperative 1, 2, 4, 6, and 10 weeks. One week postoperatively, the elbow was immobilized with a cast under neutral rotation and 90° of flexion. The K-wires were removed 4 weeks after surgery. After the pin removal, passive and active exercises for the range of motion of the elbow was encouraged.

Demographic and perioperative data included age, sex, injured side (right or left), type of fracture (extension or flexion), pattern of fracture (transverse or oblique), type of modified Gartland fracture, preoperative nerve palsy, Pucker sign, time from injury to operation, method of operation (closed reduction and percutaneous pinning [CRPP] or open reduction and percutaneous pinning [ORPP]), operative time, and follow-up period. The type of modified Gartland fracture consisted of type I, II, IIa, IIIb, and IV [14,15,16].

**Figure 1:**
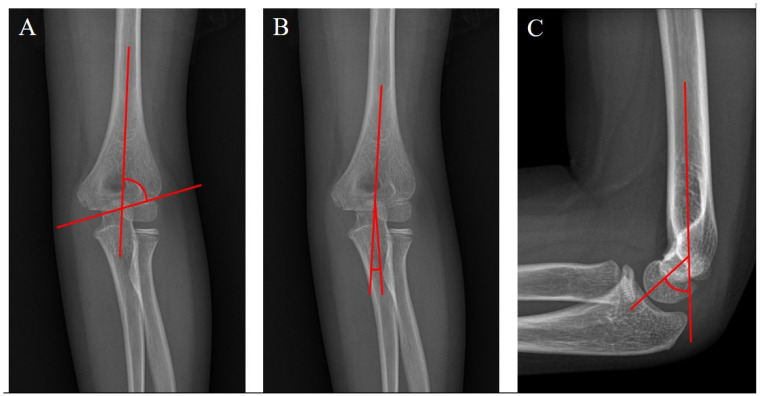
Angles for the evaluation of radiologic outcomes. (**A**) The Baumann angle was measured using the line parallel to the lateral condylar physis and the line perpendicular to the axis of the humeral shaft on AP radiographs. (**B**) The ulnohumeral angle was assessed using the ulnar and humeral length axes on AP radiographs. (**C**) The humerocapitellar angle was measured using the humeral length axis and the line parallel to the capitellum on lateral radiographs.

**Figure 2:**
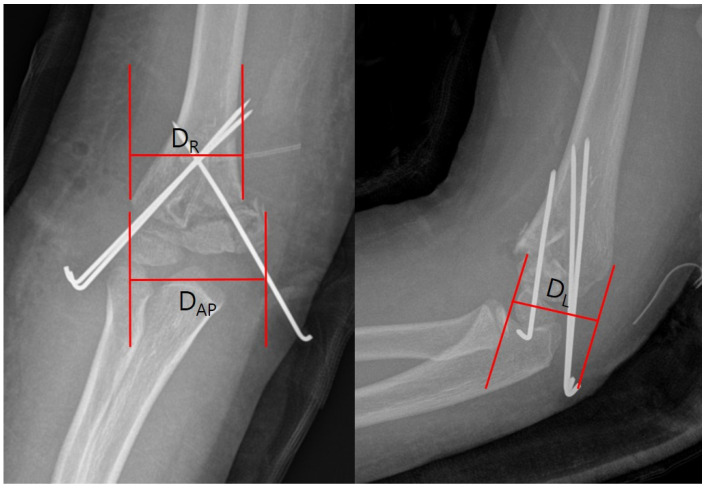
Measurements and equation of rotational angle. The dimensions of the distal fracture line (D_AP_) and the proximal fracture line (D_R_) were measured on AP radiographs. The distal fracture line (D_L_) was also measured on lateral radiographs. The rotational angle was calculated using the following formula: rotation angle = arc cos [(D_R_ − D_L_)/(D_AP_ − D_L_)].

Anteroposterior (AP) and lateral radiographs were taken before and immediately after surgery. Thereafter, AP and lateral radiographs were obtained during each outpatient clinic visit. The preoperative, immediately postoperative, and final follow-up radiographs were evaluated. Image files obtained through the INFINIT program (INFINITT, Seoul, Republic of Korea) from the high-resolution medial picture archiving communication system (PACS; IMPAX, Agfa Healthcare, Mortsel, Belgium) were used. The Baumann angle was measured using a line parallel to the lateral condylar physis and a line perpendicular to the axis of the humeral shaft on AP radiographs (Figure 1A). The ulnohumeral angle was assessed using the ulnar and humeral length axes on AP radiographs (Figure 1B). The humerocapitellar angle was measured using the humeral length axis and a line parallel to the capitellum on lateral radiographs (Figure 1C). The rotational angle was assessed using Henderson’s method [17]. The dimensions of the distal fracture line (D_AP_) and proximal fracture line (D_R_) were measured on AP radiographs. The distal fracture line (D_L_) was also measured on lateral radiographs (Figure 2). The rotational angle was calculated using the following formula: rotation angle = arc cos [(D_R_ − D_L_)/(D_AP_ − D_L_)] [14]. If the immediately postoperative rotational angle was five degrees or higher, it was defined as an immediate postoperative rotational deformity.

The clinical outcome was assessed by the recovery of range of motion of the elbow. Recovery of range of motion was defined as extension > 10° of hyperextension and flexion > 140°. The duration of recovery of range of motion was compared between the two groups. The Flynn criteria were assessed based on the loss of elbow movement during the final follow-up period [18]. Among the 88 patients, long-term clinical outcomes were evaluated for those who had progressed for more than 5 years after the operation. Long-term clinical outcomes were measured using the Oxford elbow score through a telephone survey.

In order to assess the reliability of the radiologic measurements, two authors (BSK and YGP) who were orthopedic surgeons evaluated the radiologic measurements three times at an interval of one week. Interobserver and intraobserver reliabilities of measurements were assessed using the intraclass correlation coefficient (ICC) of the radiographic measurements, and an agreement of 0.75 was considered excellent. Continuous data are presented as means and standard deviations (SD), and categorical data are presented as frequencies or proportions. The Student’s *t*-test was used for continuous data, and the chi-square test was used for categorical data. Recovery of the full range of motion of the elbow was analyzed using Kaplan–Meier survival curves. All analyses were performed using SPSS (version 24.0; IBM Corp., Armonk, NY, USA), and *p* < 0.05 was considered to be significant.

## 3. Results

**Figure 3:**
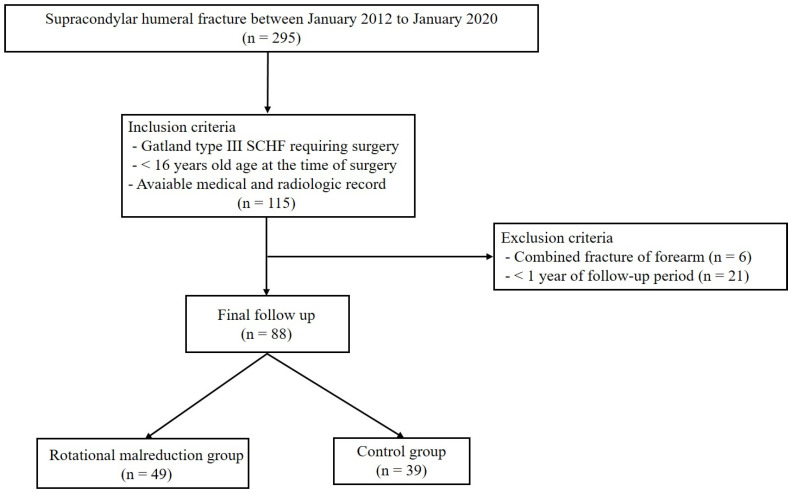
Flowchart of patient inclusion.

Among the 88 consecutive patients, 49 had immediately postoperative rotational malreduction (rotational malreduction group), and 39 had no rotational malreduction (control group) (Figure 3).

The rotational deformity group had significantly more patients with oblique fracture pattern (*p* = 0.031) and Pucker sign (*p* = 0.016) than the control group. The rotational deformity group had a significantly longer operative time than the control group (*p* = 0.029) (Table 1).

**Figure 4:**
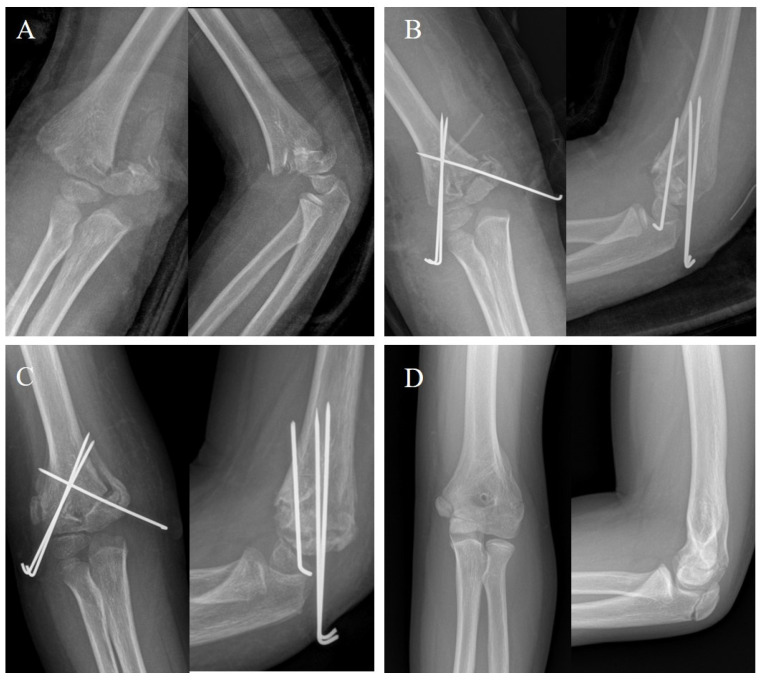
A case of an eight-year-old boy in the rotational malreduction group. (**A**) The preoperative radiographs showed modified Gartland type IIIa supracondylar humeral fractures. (**B**) The immediately postoperative radiographs showed immediately postoperative rotational malreduction. (**C**) The six-week postoperative radiographs showed the formation of calluses. (**D**) The final follow-up radiographs showed remodeling of the rotational malreduction. The patient recovered full range of motion of elbow. The Flynn criteria was excellent, and the Oxford elbow score was 100.

Each radiographic measurement showed excellent interobserver and intraobserver agreement. The preoperative humerocapitellar angle of patients with immediately postoperative rotational malreduction was significantly larger than that of control patients. However, the immediate humerocapitellar angle was not significantly different between the two groups. The preoperative and immediate rotational angles of patients with immediately postoperative rotational malreduction were significantly larger than those of control patients. However, the final rotational angle was not significantly different between the two groups (Table 2) (Figure 4).

**Figure 5:**
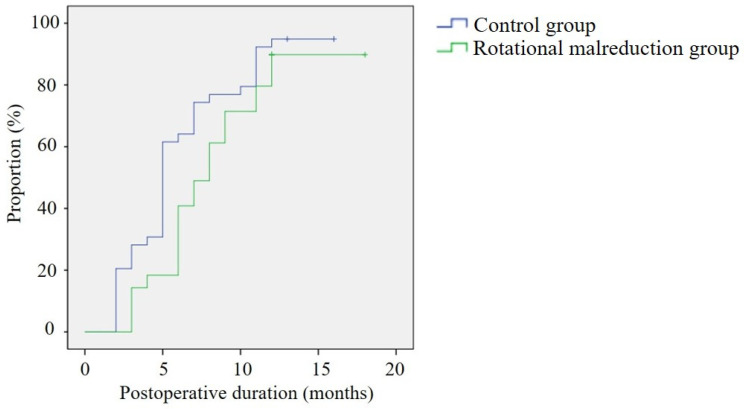
Kaplan–Meier survival curve showing number of months required to recover the full range of motion of the elbow between the rotational reduction group and control group.

Although there was no significant difference in the range of motion of the elbow and the Flynn criteria between the two groups, the Kaplan–Meier survival curve showed that the rotational malreduction group required a longer time to recover the full range of elbow motion (*p* = 0.040) (Figure 5).

Thirty patients were present in the rotation deformity group five years after surgery. Among the 30 patients, 22 responded to the Oxford elbow score questionnaire through a telephone survey. Twenty-five patients were present in the control group five years after surgery. Among the 25 patients, 19 responded to the Oxford elbow score questionnaire through a telephone survey. There was no significant difference in the long-term clinical outcomes assessed using the Oxford elbow score between the two groups (Table 3).

## 4. Discussion

This retrospective case–control study investigated the long-term clinical outcomes and associated factors for immediately postoperative rotational malreduction in the axial plane in SCHF. The immediately postoperative rotational malreduction was associated with an oblique fracture pattern and Pucker sign. Although the operative time and time to recover the full range of elbow motion was longer in immediately postoperative rotational malreduction patients, long-term clinical outcomes were favorable.

The immediately postoperative rotational malreduction was associated with an oblique fracture pattern. The Gartland classification is primarily used to describe the severity of SCHF in the coronal and sagittal planes [19]. However, the Gartland classification cannot describe the fracture pattern, obliquity of the fracture line, or comminution of the fracture [20]. In SCHF, the oblique fracture pattern can be translated into shear forces and cannot be stabilized after K-wire fixation [21]. Moreover, the rotational shearing force of the fracture can lead to a varus malalignment of the distal fragments [11]. Shah et al. reported that substantial obliquity (coronal obliquity > 10°) of the SCHF tends to heal with radiographic malunion with a significantly higher incidence [20]. If the fracture pattern of SCHF is oblique, immediately postoperative rotational malreduction should be considered. Moreover, in the case of an oblique fracture pattern, the surgeon should prepare to perform other maneuvers such as open reductions or stable fixation constructs, such as two crossed pins placed from medial and lateral condyles, or stable lateral entry pin fixations to better manage reduction [22,23].

The immediately postoperative rotational malreduction was associated with the Pucker sign. The Pucker sign is a skin dimpling at the antecubital fossa. It occurs when the proximal fragment of the SCHF penetrates the brachialis muscle [24]. The presence of the Pucker sign is associated with soft tissue injury, including median nerve and brachial artery entrapment [25]. Moreover, severe displacement and difficulty in reducing fractures are expected [26]. For this reason, immediately postoperative rotational malreduction was associated with the Pucker sign.

The rotational malreduction group had a significantly longer operative time than the control group. The classical reduction methods of SCHF in children are initial longitudinal traction and correction of the varus-valgus angulation. Hyperflexion of the elbow is successful in hyperextension deformities [27]. However, correction of the rotational deformity may be difficult with the classical reduction method because it can hardly represent the rotational force [28]. Several methods have been introduced to correct the rotational deformities. Turgut et al. reported an open procedure from the lateral aspect of the elbow and rotation of the proximal fragment using a reduction clamp [28]. Novais et al. described a method in which a Kirschner wire was inserted into the distal fragment as a joystick to correct the rotational deformity [29]. It is believed that the operative time of the rotational malreduction group was longer because rotational deformity is not reduced by classical reduction methods and requires an additional method.

Moreover, an immediately postoperative rotational malreduction may limit elbow flexion [10]. Because of this, the rotational deformity group may have required a longer time to recover the full range of elbow motion. However, rotational deformity is absorbed and remodeled with growth, and this limitation is temporary [11]. As a result, there was no significant difference in the range of motion of the elbow and the Flynn criteria between the two groups.

Although there was an immediately postoperative rotational malreduction, the short-term clinical outcome assessed by the Flynn criteria and the long-term clinical outcome assessed by the Oxford elbow score were not significantly different than the control group. Several studies have reported clinical outcomes of rotational malreduction after SCHF surgery. Gedikbas et al. reported that rotational malreduction may be associated with cubitus varus deformity, and emphasized the need for intraoperative assessment to avoid long-term deformity and cosmetic problems [30]. However, Shin et al. confirmed that there was no association between rotational malreduction and cubitus varus deformity [13]. Greve et al. reported that rotational malreduction was not associated with cubitus varus deformity and did not result in poor long-term clinical outcomes [11]. The clinical outcomes of the rotational malreduction of SCHF are still controversial. Further studies with larger sample sizes are required to verify the association between rotational malreduction and clinical outcomes in SCHF.

This study had several limitations. This was a retrospective study with a small sample size. Although long-term clinical outcomes were assessed using the Oxford elbow score, many patients were not followed-up. Moreover, the Oxford elbow score was not validated for pediatrics.

## 5. Conclusions

The oblique fracture pattern of SCHF could be associated with immediately postoperative rotational malreduction in the axial plane after operative treatment. Although there was no significant difference in long-term clinical outcomes, patients with immediately postoperative rotational malreduction required more weeks to recover the full range of elbow motion.

## Figures and Tables

**Table 1:** Demographic data and perioperative data between rotational malreduction group and control group.

	Rotational Malreduction Group	Control Group	*p*
No. of patients	49	39	
Age (mean ± SD)	5.7 ± 2.3	5.7 ± 2.3	
<5	18	12	0.695
≥5, <10	25	27	0.128
>10	6	0	
Sex, n (%)			0.638
Male	27 (55.1)	24 (61.5)	
Female	22 (44.9)	15 (38.5)	
Side, n (%)			
Right	21 (42.9)	15 (38.5)	1.000
Left	28 (47.1)	24 (61.5)	
Type of fracture, n (%)			
Extension type	46 (93.9)	38 (97.4)	0.552
Flexion type	3 (6.1)	1 (2.6)	
Pattern of fracture, n (%)			
Transverse	31 (63.3)	34 (87.2)	0.031
Oblique	18 (36.7)	5 (12.8)	
Gartland type of fracture n (%)			0.421
III-a	27 (55.1)	27 (69.2)	
III-b	19 (38.8)	12 (30.8)	
IV	4 (8.1)	0 (0.0)	
Preoperative nerve palsy, n (%)	9 (18.4)	4 (10.3)	0.309
Pucker sign, n (%)	5 (10.2)	0 (0.0)	0.016
Time from injury to operation			0.210
<24	31 (63.3)	27 (69.2)	
≥24, <48	11 (22.4)	5 (12.8)	
>48	8 (16.3)	7 (17.9)	
Method of operation, n (%)			0.116
CRPP	46 (93.9)	39 (100.0)	
ORPP	3 (6.1)	0 (0.0)	
Operative time (mins)	42.0 ± 24.7	31.7 ± 16.3	0.021
Follow-up period (months)	15.5 ± 4.0	16.6 ± 4.2	0.347

Continuous values are presented as mean and standard error (mean ± SD); categorical parameters are presented as count with percentage (%). CRPP, close reduction and percutaneous pinning; ORPP, open reduction and percutaneous pinning.

**Table 2:** Radiologic outcome between delirium patients and non-delirium patients.

	Rotational Malreduction Group	Control Group	*p*
Baumann angle (°)			
Preoperative	71.1 ± 13.2	76.3 ± 12.1	0.069
Immediately postoperative	75.2 ± 7.5	75.2 ± 6.3	0.983
Final	75.7 ± 6.2	74.9 ± 3.9	0.458
Ulnohumeral angle (°)			
Preoperative	15.1 ± 9.6	13.8 ± 6.3	0.462
Immediately postoperative	16.3 ± 9.9	15.2 ± 9.6	0.385
Final	11.6 ± 5.7	12.8 ± 5.1	0.365
Humerocapitellar angle (°)			
Preoperative	19.2 ± 15.4	31.1 ± 25.2	0.045
Immediately postoperative	42.2 ± 12.1	41.2 ± 11.6	0.475
Final	39.6 ± 11.9	40.3 ± 13.3	0.819
Rotational angle (°)			
Preoperative	41.2 ± 20.2	26.2 ± 14.8	0.004
Immediately postoperative	25.8 ± 11.5	0.4 ± 1.1	<0.001
Final	3.3 ± 6.7	0.0 ± 0.0	0.275

Continuous values are presented as mean and standard error (mean ± SD).

**Table 3:** Clinical outcome between delirium patients and non-delirium patients.

	Rotational Malreduction Group	Control Group	*p*
Restoration of full ROM			
achievement rate, n (%)	44 (89.8)	37 (94.9)	0.456
Time duration (months)	7.8 ± 3.4	6.1 ± 3.7	0.018
Flynn criteria, n (%)			0.458
Satisfactory	44 (89.8)	37 (94.9)	
Excellent	34 (69.4)	29 (78.4)	
Good	10 (20.4)	8 (20.5)	
Unsatisfactory	5 (10.2)	2 (5.1)	
Fair	2 (4.1)	1 (2.6)	
Poor	3 (6.1)	1 (2.6)	
Oxford elbow score	94.5 ± 4.5	96.4 ± 3.7	0.684

Continuous values are presented as mean and standard error (mean ± SD); categorical parameters are presented as count with percentage (%). ROM, range of motion.

## Data Availability

The data presented in this study are openly available.
